# Special Issue “Molecular Structure, Electronic, and Vibrational Spectra Theoretical Calculations in Materials Sciences”

**DOI:** 10.3390/ijms26167941

**Published:** 2025-08-18

**Authors:** Georgiy V. Girichev

**Affiliations:** Department of Physics, Ivanovo State University of Chemistry and Technology, Sheremetevsky Avenue 7, 153000 Ivanovo, Russia; g.v.girichev@mail.ru; Tel.: +7-4932-35-9874

## 1. Introduction

The dialectic of the development of science is expressed in the fact that today’s experiments must give way to tomorrow’s theory, which makes these experiments unnecessary, but which, in turn, must give way to a new, more accurate experiment the day after tomorrow, and so on along the path of progress. In the case of structural studies, the problem along this path is the different physical meaning of the molecular parameters obtained in the experiment and in quantum chemical calculations, where the researcher is faced with the task of comparing them with each other or using the calculated data to explain the properties of a real object.

Experimental data refer to a real physical object. For example, structural parameters, obtained by diffraction methods, are averaged over electronic, vibrational, and in some cases rotational levels populated under the experimental conditions, or they bear the imprint of collective interaction. The latter often manifests itself in the results of XRD and many spectroscopic studies.

At the same time, the structural and spectroscopic parameters obtained in routine quantum chemical calculations refer to the unobservable hypothetical equilibrium structure of the molecule. In this regard, the problem of comparing and mutually complementing experimental and calculated data, which are in principle incompatible due to different physical meanings, arises, the solution of which requires further development.

Currently, the most important technique for theoretical calculations in the field of chemistry and materials science is the use of quantum chemical methods of varying levels of complexity.

Quantum chemistry in its development appears in the form of stages associated with both the improvement of the theoretical level and the expansion of the range of research objects. The beginning of quantum chemistry as a special branch of physical chemistry was laid thanks to the concept of the French physicist Louis de Broglie, according to whom the key elementary particle responsible for the chemical properties of a substance, the electron, has both corpuscular and wave properties; this was presented in his article [1] (this phenomenon subsequently received a special name—wave–particle duality). The article mentioned was published in 1925, and this year marks exactly 100 years since its publication. Based on the concept of Louis de Broglie, the Austrian physicist Erwin Schrödinger in his classic works [2,3] formulated the so-called the wave equation that bears his name and describes the state of an electron in an atom; namely, it became the basis of quantum chemistry and retains its significance to this day.

In its development, quantum chemistry has gone through a number of stages, expanding its application from the analytical (theoretical) description of the object of study to its numerical modeling, consistently increasing the theoretical level of description of the system under study.

By now, two main approaches to calculating molecular characteristics have been formed—*ab initio* methods and DFT methods. The latter methods are less demanding on computer resources for calculations, and they have today received a significantly larger scale of practical application than *ab initio* methods—Hartree–Fock and post-Hartree–Fock methods. Nevertheless, the “gold standard” of quantum chemistry for small molecules is the CCSD(T) coupled cluster method, which belongs to the *ab initio* method class.

Returning to the beginning of this article, which generally examines the interaction of experiment and theory as the basis of the dialectic of scientific development, it is important to note the main pragmatic purpose of theory—replacing an expensive experiment while simultaneously ensuring the accuracy of predicting the properties being studied, which is not inferior in accuracy to experimental data.

At present (if not in principle at all), the key to the successful development of theoretical methods is the presence of metrologically reliable and clear in physical meaning experimental data, which can be relied upon when testing new theoretical constructs.

Among spectroscopic methods, microwave spectroscopy is most widely used to determine the exact geometric structure of free molecules. However, the application of this method is limited to molecules consisting of a relatively small number of atoms and possessing a dipole moment.

A valuable method for establishing molecular structure is NMR. However, the accuracy of determining internuclear distances through this method is 1–2 orders of magnitude lower than the accuracy of microwave and diffraction methods. It should be noted that the obtained structural data, as a rule, relate to the condensed state of the substance or solution and largely concern clarifying issues of the chemical identification of the sample, conformational analysis, internal influence effects, etc.

Valuable information about the geometric and electronic structure of molecules can be obtained by studying their vibrational (IR and Raman) and electronic (emission, absorption, etc.) spectra.

The most frequently used method for determining the structure of substances in the solid state is single-crystal XRD. If the crystal is molecular, the geometric structure of separate molecules can be found. Thanks to favorable statistics, namely, a very wide sample (the number of reflections recorded during X-ray structural analysis of single crystals can reach tens of thousands), this method allows us to determine interatomic distances with very high metrological accuracy. However, compared to the structure of an isolated (individual) molecule, the molecular structure obtained in X-ray diffractometry is distorted by the collective (intermolecular) interaction in the crystal. This distortion can significantly exceed the error of the diffraction experiment and in some cases can lead to erroneous conclusions when using X-ray structural data to describe the steric and electronic effects that determine the structure of an isolated molecule. Another factor that must be considered when using most XRD structural data is that the scattering of X-rays occurs on the electron shells of atoms in a molecule. Therefore, it is not the internuclear distances that are determined, but the distances between the centers of gravity of the electron density on the atoms, which can differ significantly from the internuclear ones due to the specifics of the chemical bond that is formed. Examples of such situations are the distances between light atoms that have lone electron pairs or the distances between atoms, one of which is a hydrogen atom. In the latter case, special geometric techniques are used that formally define the positions of hydrogen atoms.

The listed circumstances should be taken into account when formulating conclusions based on a comparison of the XRD and quantum chemical structure.

The main experimental method for determining the structure of polyatomic (up to 100 atoms or more) molecules free from collective interaction is gas electron diffraction. However, the molecules under study must be transferred to the gas phase. In some cases, the vaporization process is accompanied by the incongruent evaporation of the sample or oligomerization, its interaction with the inlet system material, and in some cases this requires an effusion experiment at elevated temperatures (up to 2000 K and higher), which significantly complicates both the experiment and the interpretation of its results. The issue of identifying the object under study was resolved in the works [4,5], in which the authors combined electron diffraction and mass spectrometric experiments, thereby obtaining the ability to continuously monitor the composition of the vapor under study throughout the diffraction experiment. However, the different physical meaning of the structure obtained by electron diffraction (it is a nuclear configuration averaged over all electronic, vibrational and rotational states populated under the experimental conditions) and the structure from quantum chemical calculations (it corresponds to a minimum on the potential energy surface and is unobservable in the experiment) greatly complicates the task and requires that the specifics of intramolecular dynamics, based on the calculated quantum chemical force field of the molecule, are taken into account when deciphering the diffraction pattern of molecules.

Considering the vibrational effects and the effects of centrifugal distortion when interpreting the data of a gas-phase electron diffraction experiment allows us to determine the equilibrium molecular structure, which in its physical meaning coincides with the calculated quantum chemical structure. This further allows us to use it equally with the calculated structure to establish structure–property correlations of real objects or when testing quantum chemical methods for calculating molecular structure.

Thus, the question of the physical meaning of the obtained molecular characteristics is in many cases the most crucial key if we want to explain the subtle effects of the relationship between the structure and physicochemical properties of substances.

This introduction does not claim to be an exhaustive examination of all methods capable of providing information on the structure of molecules, but it merely draws the attention of researchers to the validity and quantitative side of conclusions that are based on a comparison of the structural and energy characteristics of molecules obtained by different methods.

This Special Issue “Molecular structure, electronic and vibrational spectra. Theoretical calculations in materials science” represents a characteristic cross-section of the current situation in the literature, with the help of which one can indirectly conceptualize the nature of the interaction of experiment and theoretical calculations and the direction of development of the problem under discussion.

## 2. An Overview of the Published Articles

Contribution 1. The work of Adabekyan and co-authors is a classic example of the successful complementarity of experimental and computational methods for the purpose of deep penetration into the physicochemistry of the compounds studied. In this work, the photoprocesses in 1,4-diazadistyrylbenzene and 1,3-diazadistyrylbenzene derivative diperchlorates in MeCN were studied by absorption, luminescence, and kinetic laser spectroscopy. The comprehensive experimental measurements were added by DFT/PBE0/6-31+G(d,p)/PCM(MeCN) calculations of *cis* and *trans* isomer structure diazadistyrylbenzenes and their electrocyclization products (dihydrophenanthrenes DHP-1 and DHP-2) in the ground S0 and singlet excited S1 states. The absorption and emission spectra were calculated by TDDFT with the same functional, basis set, and solvent model. The information obtained in the work on photoprocesses in diazadistyrylbenzenes can be implemented in the construction of photoactive supramolecular systems based on their use of macrocyclic compounds.

Contribution 2. The work of Giricheva and co-authors has demonstrated an even closer connection between experimental and theoretical methods. The main goal of the work was to establish the possibility of the existence of cyclic dimers of 4-n-propyloxybenzoic acid formed by hydrogen bonds in the gas phase. The authors used a combined method, including gas electron diffraction, mass spectrometry, and DFT calculations. The calculated structural parameters were refined during the LS structural analysis of the experimental diffraction pattern. In addition, the calculated force field was used both to calculate the starting values of the root mean square vibration amplitudes and vibrational corrections to internuclear distances in order to give them the physical meaning of values close to equilibrium ones. Thus, the experimental and calculated structural parameters were reduced to a single scale, thereby ensuring their correct comparison. The energy of hydrogen bonds to the cyclic fragment of the found dimer was calculated quantum chemically and the bonding nature was described within the framework of NBO analysis.

Contribution 3. The work of Sagan and co-authors is a completely theoretical study of chemical bonding in the associates “cyclopropenylidene—MX_2_” and “imidazol-2-ylidene—MX_2_”, where M = Be, Mg, Zn; X = H, B. The physical nature of these “carbene…MX_2_” interactions has been quantitatively characterized by the joint use of a topological QTAIM-based Interacting Quantum Atoms (IQA) decomposition scheme, the molecular orbital-based Extended Transition State—Natural Orbitals for Chemical Valence (ETS-NOCV) charge, and a Local Energy Decomposition scheme (LED) based on the Domain-based Local Pair Natural Orbital Coupled Cluster with Single, Double, and perturbative Triple excitations (DLPNO-CCSD(T)) method, which was used to minimize the error introduced by the theoretical level of calculations.

The characteristic feature of the considered associates is the bent structure of the MX2 molecule. The formation of the C…M bond leads to the significant elongation of the M–X bond and a greater opening of the L–C–L angle in the carbene molecule. It is also worth noting that for the bond length M–X, the relation Be < Zn < Mg applies so that for a given X, the magnesium atom forms the longest M–X bond.

Contribution 4. The work of Tverdova and co-authors reports the results of studying the geometric structure, vibrational frequencies, electronic characteristics, and thermodynamic characteristics of conformers, as well as the structure and energy of transition states of 4-(4-tritylphenoxy)phthalonitrile (TPPN), which can be used in material science as a precursor in the synthesis of phthalocyanines with bulky substituents. A set of complementary methods include gas electron diffraction, mass spectrometry, IR spectroscopy, and quantum chemical calculations (DFT/B3LYP, PBE, CAM-B3LYP, and B97D). It was found that the molecule TPPN has four conformers. All vibrational bands in the experimental IR spectrum of TPPN have been assigned by symmetry and the distribution of normal vibration energy over internal coordinates. A slight difference was noted in the theoretical IR spectra of the conformers, which, however, made it possible to determine that TPPN consists of a mixture of conformers in the condensed state. The structure of transition states between conformers is determined. The close calculated values of the total energy of the conformers and high transition barriers suggest that the conformational diversity of TPPN is formed during the synthesis of the compound.

Contribution 5. The work of Tiunova and co-authors describes the synthesis of cationic Mn(III) complexes with ligands 5-Hal-sal2323 (Hal = Cl, Br) and a paramagnetic divalent counterion [ReCl_6_]^2−^ as follows: [Mn(5-Cl-sal_2_323)]_2_[ReCl_6_] (1) and [Mn(5-Br-sal_2_323)]_2_[ReCl_6_] (2). The crystal structures and magnetic properties of both isostructural two-component ionic compounds were studied at six temperatures (from 100 to 423 K). The phenomenon of different spin behavior of Mn atoms in stoichiometrically identical fragments of [Mn(5-Cl-sal2323)] within 1 and 2 was discovered. A thermally induced spin transition at high temperature, associated with the cationic moiety, and a field-induced slow magnetic relaxation of magnetization at cryogenic temperature, associated with the anionic moiety of the complexes, were detected.

Contribution 6. In the work of Merkel and co-authors, FTIR spectroscopy in combination with DFT/B3LYP/6-311 G (p, d) calculations were applied to study the geometrical structure and intermolecular interaction of two mesogenic groups based on cyanobiphenyl (CB) with thioether-linking groups (C-S-C) termed CBSC_n_SCB (n = 5, 7) and the asymmetric system with the ether- and thioether-linking groups, termed CBSC_n_OCB (n = 5, 7). Changes in the behavior of intermolecular interactions were observed by significant differences in the values of transition dipole moments for selected vibrational bands. One significant finding of this study was evidence that the intermolecular forces evolved on the transition from the nematic phase to the twist–bend phase. A model for the packing of the dimer molecules in the twist–bend phase was developed due to the overlapping of the rigid cyanobiphenyl cores stabilizing this phase and thus affecting the helical pitch.

Contribution 7. In the work of Ferjani and co-authors, new salt of the antifungal drug fluconazole, (H_2_Fluconazole)·SnCl_6_·2H_2_O, was synthesized and characterized by single-crystal XRD, Hirshfeld surface analysis, Raman, FTIR, UV-visible spectroscopies, and magnetic property measurements. To understand and predict the chemical behavior of (H_2_Fluconazole)·SnX_6_·2H_2_O; (X = F, Cl, Br, and I), global chemical reactivity descriptors were used, including E_HOMO_, E_LUMO_, ionization potential, electron affinity, hardness, softness, chemical potential, electrophilicity, and electronegativity, and these were calculated at the theory level HSE1PBE/6-311++G**,LANL2DZ(Sn). TDDFT calculations were performed at the optimized geometries using the same levels of theory.

Contribution 8. In the work of Loska and co-authors, FTIR spectroscopy and DFT/B3LYP/6-311 G (p, d) calculations have been used to study the structure and molecular interactions in the nematic and twist–bend phases of symmetrical and asymmetrical dimers with the cyanobiphenyl mesogenic groups linked in two mesogenic cores by methylene, thioether, and ether fragments. The presence of conformers and the knowledge of their structural and energetic characteristics are important for calculating the orientation order in liquid crystals. In this connection, the dihedral angle distributions and conformations in terms of potential energy functions for torsional motion were the objects of special attention. Another distinctive feature of this work is the presence of detailed data on the theoretical and experimental frequencies, dichroism values, relative intensity, direction of the transition dipole moment, and approximate band assignments for all dimers investigated.

Contribution 9. In the work of Qi Gong and Guiling Zhang, a state-of-the-art quantum mechanics-based program, CASTEP module of Materials Studio 2020 package (Accelrys Inc., Materials Studio version 2020.08, San Diego, CA, USA), specifically designed for solid-state materials science was applied to study of new 2D topological insulators (TIs) with a large bulk bandgap. It was identified through first-principles calculations that HSb_2_XH_n_ and HBi_2_XH_n_ form a new family of giant bandgap 2D TIs. On the examples of HSb_2_XH_n_ and HBi_2_XH_n_, spin–orbit coupling electronic structures of Janus (asymmetrically functionalized by CH_3_ or OH) Sb and Bi monolayers with nontrivial topology, Rashba splitting, and valley-contrast circular dichroism are proposed and demonstrated in the present study.

Contribution 10. In the work of Ryzhov and co-authors, the electronic and geometric structures of metal-free tetrapyrazinoporphyrazine (TPyzPA), octachlorotetrapyrazinoporphyrazine (TPyzPACl_8_), and their complexes with Al, Ga, and In were studied at the DFT/PBE0-D3/def2TZVP theory level. The main objective was to study the effect of chlorination on the structure and properties of these macrocycles.

It has been established that the structural parameters of the macrocyclic ligand undergo minimal changes when replacing a central metal atom or introducing chlorine atoms at the periphery.

The substitution of H-atoms for Cl leads to a decrease in the energy of the frontier MOs of M(Cl)TPyzPACl_8_ as compared to M(Cl)TPyzPA. The electron absorption spectrum of TPyzPACl_8_ contains three intense bands, while only two bands were found for TPyzPA. The calculated IR spectra of TPyzPACl_8_ have a significant difference in comparison with TPyzPA due to heavy Cl atoms involved in the vibrations that form medium and strong bands in the region of 1000–1500 cm^−1^.

Contribution 11. The work of Klyukin and co-authors is devoted to the combined theoretical and experimental study of the protonation of [2,6-B_10_H_8_O_2_CCH_3_]^−^. This is a typical example of the successful use of quantum chemical calculations to understand the mechanism of a chemical reaction for the synthesis of a compound, in particular, the [2,6-B_10_H_8_O_2_CCH_3_*H_fac_]^0^ complex. The structure of this complex was obtained using NMR experiments and DFT calculations. The 11B-1H NMR spectra of this complex were found to be complex and difficult to interpret. Notably, DFT/B97/IGLO-III calculations reproduced the experimental NMR spectrum well.

Contribution 12. The work of Ziyi Wang and co-authors demonstrates the effectiveness of combining experimental spectroscopy and quantum chemical calculations for interpreting vibrational spectra. The initiative of this work relates to the problem of the corrosion of technological equipment under the influence of organic sulfur compounds. However, the problem of corrosion itself is not specifically considered here, and the study is limited to a detailed analysis of the structure, as well as vibrational spectra and vibrational behaviors, of two sulfur-containing compounds, dibenzyl disulfide and dibenzyl sulfide (DBDS, DBS), as well as bibenzyl (BiBz). The obtained IR and Raman spectra of these compounds were interpreted using DFT calculations. The conformational composition was calculated according to the Boltzmann distribution in relative Gibbs free energy. The analysis of the distribution of potential energy of normal vibrations along internal coordinates was used in the assignment of experimental spectra. The Noncovalent Interaction Analysis (NCI) method was applied to reveal the nature of dominant conformations with low free energy.

No comments were made regarding the validity of the comparison of vibrational spectra, since these were measured for the solid phase and calculated for the gas.

Contribution 13. In the work of Dan Deng and co-authors, the phosphorescence mechanism of the (E)-3-(((4-nitrophenyl)imino)methyl)-2H-thiochroman-4-olate-BF_2_ compound has been theoretically investigated. The calculated radiative and nonradiative rate constants confirm the predominant relaxation scheme of the excited state S2 in the form of a chain of transitions S0→S2→S1→T2→S0, indicating a violation of the Kasha rule.

## 3. Conclusions

The articles presented in this Special Issue attest to the increasingly deep complementarity of experiments and quantum chemical calculations. In some cases, calculations act as an independent research method, claiming to eliminate the need for experiment and demonstrating the availability of obtaining the wider spectrum of properties of the compounds studied compared to experiments. Improvements in calculation methods eventually eliminate the need for an experiment in cases where this replacement cost is effective. In science circles, the following quote is known: “There is nothing more practical than theory”. However, it is necessary to remember that the effectiveness of theoretical methods is tested by comparing their predictions with the results of experiments. In this regard, the following quote of Nobel Prize Laureate, Professor Pyotr L. Kapitsa, is substantiated: “Theory is a good thing, but well done experiment lasts forever” [6]. In this regard, works that contain experimental and theoretical results that are both complementary and mutually verifiable are of particular value.

## Data Availability

Not applicable.
